# Venous Thromboembolism in Cancer: An Update of Treatment and Prevention in the Era of Newer Anticoagulants

**DOI:** 10.3389/fcvm.2016.00024

**Published:** 2016-07-28

**Authors:** Waqas Qureshi, Zeeshan Ali, Waseem Amjad, Zaid Alirhayim, Hina Farooq, Shayan Qadir, Fatima Khalid, Mouaz H. Al-Mallah

**Affiliations:** ^1^Department of Internal Medicine, Division of Cardiovascular Epidemiology and Cardiology, Wake Forest University, Winston Salem, NC, USA; ^2^Department of Internal Medicine, University of Maryland, Baltimore, MD, USA; ^3^Allama Iqbal Medical College, Lahore, Pakistan; ^4^Department of Internal Medicine, Henry Ford Hospital, Wayne State University, Detroit, MI, USA; ^5^Rawalpindi Medical College, Rawalpindi, Pakistan; ^6^Khyber Medical College, Peshawar, Pakistan; ^7^Department of Internal Medicine, Division of Nephrology and Hypertension, Wake Forest University, Winston Salem, NC, USA; ^8^King Abdulaziz Medical Center, Riyadh, Saudi Arabia

**Keywords:** venous thromboembolism, risk stratification, newer oral anticoagulants, thromboprophylaxis, low molecular weight heparin, apixaban, rivaroxaban

## Abstract

Cancer patients are at major risk of developing venous thromboembolism (VTE), resulting in increased morbidity and economic burden. While a number of theories try to explain its pathophysiology, its risk stratification can be broadly done in cancer-related, treatment–related, and patient-related factors. Studies report the prophylactic use of thrombolytic agents to be safe and effective in decreasing VTE-related mortality/morbidity especially in postoperative cancer patients. Recent data also suggest the prophylactic use of low molecular weight Heparins (LMWHs) and Warfarin to be effective in reducing VTEs related to long-term central venous catheter use. In a double-blind, multicenter trial, a new ultra-LMWH Semuloparin has shown to be efficacious in preventing chemotherapy-associated VTE’s along with other drugs, such as Certoparin and Nadoparin. LMWHs are reported to be very useful in preventing recurrent VTEs in advanced cancers and should be preferred over full dose Warfarin. However, their long-term safety beyond 6 months has not been established yet. Furthermore, this paper discusses the safety and efficacy of different drugs used in the treatment and prevention of recurrent VTEs, including Bemiparin, Semuloparin, oral direct thrombin inhibitors, parenteral and direct oral factor Xa inhibitors.

## Key Points

◦ Risk factors for venous thromboembolism can be grouped into three broad categories: cancer-related, patient-related, and treatment-related factors.
◦ Prophylactic use of anticoagulants is safe and efficacious in preventing VTE.
◦ LMWHs prove to be a good treatment option for VTE in advanced cancers, being simpler and more efficacious in preventing recurrence.

## Introduction

Cancer continues to pose a costly and growing international threat toward modern day society. Among its many direct and indirect complications is its role as a major risk factor for venous thromboembolism (VTE), discovered in a fifth of all cancer patients and as many as half on postmortem examination (1, 2). Such VTE events include, but are not limited to, central venous catheter (CVC)-related thrombosis and pulmonary embolism (PE) (3, 4). It was Professor Armand Trosseau who first described the association between cancer and thrombosis in 1865, almost 150 years ago, yet its exact pathophysiology remains poorly understood. Cancer-associated VTE bears several clinical and economic implications, including increased hospitalization rates, the need for anticoagulation (and its associated bleeding complications), in addition to the risk for recurrent VTE and the potential for delays in cancer therapy (5). This article presents an overview of VTE risk assessment in cancer patients, current treatment guidelines and the role of newer anticoagulants in the treatment of cancer-related VTE.

### Disease Burden and Economic Implications

To the practicing clinician, cancer remains the most significant acquired risk factor for the development of VTE, with an annual incidence of 1 in 200, ultimately affecting at least 15% of this population (6). VTE in patients with underlying malignancy as opposed to those without cancer can be particularly more serious given their increased likelihood of VTE recurrence, risk of major bleeding complications from anticoagulants, and their reduced survival from such events. Prandoni et al. for instance, reported that patients with cancer and VTE were approximately four times more likely to develop recurrent thromboembolic complications and twice as likely to develop major bleeding while on anticoagulation when compared to patients without underlying malignancy (7). In a retrospective analysis, Khorana et al. found that inhospital mortality was two- to fivefold higher among neutropenic cancer patients hospitalized with thromboembolism as compared to those without thromboembolism. Chew and colleagues analyzed the effect of VTE on survival between cancer patients and found that a diagnosis of thromboembolism was associated with reduced survival rates during the first year, regardless of the type of cancer studied [hazard ratio (HR) 1.6–4.2, *P* > 0.1] (8). In addition to its human cost, VTE in cancer patients confers additional economic burden. Of cancer patients who develop deep vein thrombosis (DVT), the mean cost of hospitalization in 2002 was US $20,065 (9) compared to an average between $7712 and $10,804 for a similar episode in the general population (10).

### Pathophysiology

While the exact mechanism of VTE in cancer patients is unclear, several theories seem to bear credence. It is recognized, for example, that tissue factor (TF) (which initiates the coagulation cascade) is itself expressed in a variety of malignancies and released into the circulation, suggesting its potential role in cancer-related VTE (8). One study was able to demonstrate a consistent relationship between cell surface expression of TF and prothrombotic potential across a range of sites, including breast, colorectal, and pancreatic tumor cell lines (11). Other observations have lent themselves to other theories. Falanga and Gordon (12), for example, described a cysteine protease that directly activates factor X in the absence of factor VII while Denko and Giaccia had proposed that tumor cell hypoxia stimulates production of procoagulant and angiogenic factors (13). Other theories based on animal models have raised the possibility of oncogene activation to explain the manifestations of Trousseau’s syndrome (14). Yet others describe release of mucins and their interaction with L- and P-selectins particularly in patients with mucinous adenocarcinomas (15, 16). It would not be unreasonable, therefore, to assume that some of these pathways operate and overlap in ways that ultimately predispose the cancer patient to thromboembolic events.

### Risk Stratification

It is difficult to directly compare the rates of cancer-related VTE among patients as the studies vary in their study periods, the methods employed in detecting and reporting VTE, the patient populations, and their follow-up periods. Additionally, with the temporal rise in the incidence of VTE, newer studies seem to report higher rates than those that are less recent (17, 18). Nevertheless, there is broad agreement in the literature with regards to most risk factors for cancer-related VTE. These can be broadly divided into three categories: cancer-related factors, treatment-related factors, and patient-related factors.

#### Cancer-Related Risk Factors

The site of the primary tumor has been established as a risk factor for VTE in a number of studies. Specific incidence rates vary based on the clinical setting, but some of the highest rates have been described in patients with primary brain tumors (19, 20), pancreatic (21, 22), stomach (23), uterine (24, 25), and lung carcinomas (26, 27). More recent studies suggest high incidence rates of VTE in association with hematologic malignancies as well. In a large population-based case-control study, hematologic malignancies were in fact found to confer the highest risk of venous thrombosis, followed by lung and gastrointestinal cancers (28). Even among cancer patients with the same primary site, VTE rates seem to vary markedly based on grade and histology. Blom et al., for instance, showed that lung cancer patients with adenocarcinoma had a greater incidence of venous thrombosis as compared to those with squamous cell carcinoma (29). Indeed the stage of cancer is also important, with more advanced stages of cancers conferring ever increasing risk (18, 30). It appears that this risk is highest in the period immediately following cancer diagnosis. In a large case-control study, it was reported that the risk of VTE was highest in the first 3 months following the diagnosis of cancer [adjusted odds ratio (OR), 53.5; 95% confidence interval (CI), 8.6–334.3], subsiding gradually over a 15-year period to levels observed in the general population (28).

#### Treatment-Related Risk Factors

As cancer patients too often know unfortunately, the remedy can sometimes be more toxic than the malady itself. Chemotherapy is associated with a two- to sixfold increase in the risk of VTE compared to the general population and in patients starting new chemotherapy regimens, accounts for 9% of deaths (3, 31). These trends seem to be increasing over time, perhaps owing to the development of additional chemotherapeutic options. In hospitalized patients receiving chemotherapy, rates of VTE rose from 3.9 to 5.7% from 1995 to 2003, an increase of 47% (32). Some chemotherapy agents appear to confer greater risk than others. Patients with multiple myeloma receiving Thalidomide in combination with dexamethasone, for example, have DVT rates as high as 28% in some instances (33, 34). Additional predictors for Thalidomide associated VTE include its combined use with Doxorubicin (OR = 4.3), newly diagnosed disease (OR = 2.5), and Chromosome 11 abnormalities (OR = 1.8) (35). Another commonly used agent, Lenalidomide, has significant survival benefits in myeloma patients while also being associated with rates of VTE as high as 75% (36). Another agent, Bevacizumab (an anti-angiogenic in use for a variety of cancers) has been associated with increased risk of both arterial (37) as well as venous (38) events. Strategies to mitigate VTE events in such patients continue to be investigated, though intermittently dosed chemotherapy regimens appear to lessen such risks when compared to continuous treatment (39).

Even common and seemingly innocuous practices, such as the administration of erythropoiesis-stimulating agents (ESAs) to treat anemia, can be harmful in the cancer patient. In a systematic review of 57 trials on cancer patients, thromboembolic events were observed in 229 of 3,728 patients treated with Epoetin or Darbepoetin and in 118 of 3,041 untreated controls (RR = 1.7; 95% CI, 1.4–2.1) (40). In hospitalized cancer patients, it is often necessary to transfuse blood and platelet products both of which are associated with an increased risk of thromboembolic events as well as mortality (41).

While surgery and a prolonged postoperative period are well-known risk factors for VTE, recent data suggest these may not be as significant as other factors, possibly owing to increased thromboprophylaxis rates among the surgical patient population (31, 42–44). Nevertheless, cancer patients undergoing surgery are still at risk for VTE events, particularly in those >60 years of age (OR = 2.6, 95% CI, 1.2–5.7), with prior episodes of VTE (OR = 6, 95% CI, 2.1–16.8), advanced stages of cancer (OR = 2.7, 95% CI, 1.4–5.2), anesthesia lasting more than 2 h (OR = 4.5, 95% CI, 1.1 to 19), and bed rest exceeding 3 days (45).

Central venous catheters are widely used in patients with cancer for the administration of chemotherapy. Verso et al. reported that the incidence of symptomatic catheter-related DVT in adults ranges from 0.3 to 28% while that of catheter-related DVT screened by venography ranges from 27 to 66% (46). The specific chemotherapy agent administered through the catheter can also influence the risk of DVT (47). Other treatment-related risk factors for VTE that have been described in the literature include hospitalization (48, 49) and radiation (31, 50).

#### Patient-Related Risk Factors

The overall risk of VTE is often affected by a multitude of patient-related factors, including a history of prior thrombotic events, comorbid conditions, genetic factors, immobility, age, sex, and race (Table 1). Prior thrombotic episodes significantly increase future thrombotic risk in a wide range of cancer patients including those with prostate cancer, myeloma, and cancer patients undergoing surgery to name a few (OR = 6.0, 95% CI, 2.1–16.8) (51, 52). Concurrent thrombotic events, either venous or arterial are also thought to increase the risk for VTE (32). In patients with hepatocellular carcinoma, for instance, it is noted that the incidence of systemic VTE is higher in patients with concurrent portal vein thrombosis when compared to those without it (11.5 vs. 4.4%, *P* = 0.04) (53). It is suggested that locally occurring thrombotic events may propagate pathways that result in systemic hemostatic activation. In addition to obtaining a history of prior VTE events, one must elucidate details of a patient’s comorbid conditions as these are invariably apt to influence thrombotic risk. Khorana et al. for instance, showed that infections (OR = 1.77), renal disease (OR = 1.53), arterial thromboembolism (OR = 1.45), pulmonary disease (OR = 1.37), and anemia (OR = 1.35) (32) are strongly associated with VTE risk in cancer patients. Not surprisingly then, in a study on patients with ovarian cancers, Rodriguez et al. observed that the risk of VTE continued to increase with the number of such comorbid conditions; (HR = 2.1 with one comorbidity, HR = 2.6 with two comorbidities and HR = 3.9 with ≥ three comorbidities) (44).

**Table 1:** **Clinical risk factors for cancer-associated venous thromboembolism**.

**Cancer-related factors**
Primary site of cancer (17, 18, 28, 30, 32); brain, pancreas, kidney, stomach, lung, gynecologic, lymphoma, myeloma Advanced stage of cancer (18, 30, 45) Initial period after diagnosis of cancer (26, 31, 54, 55) Histology (29, 56)
**Treatment-related factors**
Major surgery (45) Hospitalization (32, 48, 49) Cancer therapy ◦ Chemotherapy (3, 31, 39, 48) ◦ Hormonal therapy (57) ◦ Anti-angiogenic agents (33–37, 58–60) ◦ Erythropoiesis-stimulating agents (40, 61) ◦ Transfusions (41) ◦ Central venous catheters (46, 47)
**Patient-related factors**
Older age (32, 45) Female sex (32) Race (32, 43, 56) ◦ Higher in African American ◦ Lower in Asians/Pacific Islanders Comorbidities (43, 44, 48, 56, 62) Inherited prothrombotic mutations (28, 63); Factor V Leiden, prothrombin gene mutation Prior h/o VTE (45, 51–53) Performance status (26, 45)
**Biomarkers** (61, 62, 64, 65)
Prechemotherapy platelet count ≥350,000 Prechemotherapy leukocyte count >11,000 Increased tumor cell expression of tissue factor (TF) (8, 21, 66)

*Modified from Khorana et al*.

Finally, one must obtain a detailed family history when assessing a patient’s thrombotic risk. This is important as a number of predisposing genetic factors, such as Factor V Leiden and Prothrombin gene mutations are known to confer an increased risk of VTE in cancer patients when compared to those without the mutations (63, 67). Even when the above-mentioned factors have been evaluated, a comprehensive VTE risk assessment in the cancer patient is incomplete until weighed in the context of their functional status. In a prospective study on patients with non-small cell lung carcinoma receiving chemotherapy, VTE developed in 31% patients with poor performance status as compared to 15% with better performance status (26).

### A Risk Model for Chemotherapy-Associated VTE

In order to reduce the disease burden of VTE, it is important to identify cancer patients at high risk for VTE and who may, therefore, benefit from thromboprophylaxis. Conversely, identifying patients at low risk for VTE may allow us to determine those patients in whom prophylactic anticoagulation can be foregone in order to mitigate the risk of iatrogenic bleeding. Though both intuitive and appealing, the utility of such an approach has had conflicting results (68–70). Recently, a validated risk model for use in the ambulatory setting was published to identify cancer patients whom are at high risk for VTE (Table 2) (62). Five predictive variables were identified in the development cohort, before initiation of chemotherapy. Rates of VTE in the development cohort were 0.8% in the low-risk category (score = 0), 1.8% in the intermediate risk category (score = 1–2), and 7.1% in the high risk category (score = 3 or above), while rates in the validation cohorts were 0.3, 2, and 6.7%, respectively (over a median period of 2.5 months, C statistics = 0.7 for both cohorts).

**Table 2:** **Predictive model for chemotherapy-associated VTE (62)**.

Patient characteristics	Risk score
Site of cancer	
*Very high risk (stomach, pancreas)*	2
*High risk (lung, lymphoma, gynecologic, bladder, testicular)*	1
Prechemotherapy platelet count ≥350,000/μL	1
Hemoglobin <10 g/dl or use of RBC growth factors	1
Prechemotherapy leukocyte count >11,000/μL	1
Body mass index ≥35 kg/m^2^	1

*High risk defined as risk score ≥3. VTE, venous thromboembolism*.

The model has a negative predictive value of 98.5% at the cutoff point for high risk (score ≥ 3). This score is being used to define cancer patients at high risk for VTE in a study on thromboprophylaxis.^1^ Additionally, this model is being assessed for its use in clinical practice as well.^2^

## Prophylaxis of Venous Thromboembolism

### Prevention of Postoperative VTE

In surgical oncology patients without thromboprophylaxis, the incidence of lower extremity DVT, as shown by venography, ranges from 40 to 80% while that of proximal DVT varies between 10 and 20%. The risk of fatal postoperative PE associated with cancer surgery is about four times higher in comparison to non-oncologic surgery (71). Strong risk factors associated with VTE in this setting have been described above (treatment-related risk factors). One commonly used prophylactic regimen consists of a pre-operative dose of subcutaneously administered Heparin, followed by scheduled dosing 12–24 h postoperatively. Typically, unfractionated Heparin (UFH) is given two to three times a day and low molecular weight Heparin (LMWH) is injected once daily. International guidelines direct the use of anti-thrombotic prophylaxis for at least 7–10 postoperative days in patients with cancer surgery (5, 72–74).

Enoxaparin and Cancer (ENOXACAN) II was the first study to demonstrate the benefits of extended thromboprophylaxis with enoxaparin in reducing postoperative VTE among cancer patients undergoing surgery (75). These benefits have been demonstrated across a wide range of patient profiles among oncologic patients, including those undergoing abdominal, pelvic, and thoracic surgery. Rasmussen et al. showed that prolonged prophylaxis with LMWH significantly reduces the risk of VTE compared with prophylaxis given for 7–10 days, without an increase in bleeding complications (76). The overall incidence of VTE was 14.3% (95% CI, 11.2–17.8%) in the control group as compared to 6.1% (95% CI, 4.0–8.7%) in the patients receiving out-of-hospital LMWH (76). The ESMO and AIOM guidelines (73, 74) recommend extended prophylaxis for all patients undergoing elective cancer surgery while the ASCO panel (5) recommends extended prophylaxis for up to 4 weeks in patients undergoing major abdominal or pelvic cancer surgery in the presence of strong risk factors (please see above).

If pharmacologic prophylaxis is contraindicated, mechanical prophylaxis, such as continued use of intermittent pneumatic compression devices with or without compression stockings, should be employed. If compression stockings are used, it is important to ensure that they are of the appropriate size in order to be effective in preventing DVT (77).

### Prevention of VTE-Related to Long-Term CVC

The incidence of asymptomatic CVC-related DVT has been reported to be about 20% while that of overt DVT of the upper extremities ranges between 2 and 4% (71). Thromboprophylaxis for CVC-related thrombosis is controversial. Both LMWH and Warfarin have been found to be safe and effective in these patients (68, 70, 78, 79). International guidelines, however, do not recommend routine prophylaxis for this indication (5, 73, 74).

### Prevention of Chemotherapy-Associated VTE

In one of the earliest studies examining this topic, Levine et al. reported that low-dose Warfarin is safe and effective for VTE prevention in stage IV breast cancer patients receiving chemotherapy, the relative risk reduction vs. placebo being 85% (80). In the more recent PROTECHT study, Nadroparin use was found to be associated with a statistically significant 50% relative risk reduction in thromboembolic events among cancer patients in the ambulatory setting receiving chemotherapy for advanced cancers of the breast, lung, gastrointestinal tract, head/neck region, ovary, and pancreas. Fifteen (2.0%) of 769 patients treated with Nadroparin and 15 (3.9%) of 381 patients treated with placebo had a thromboembolic event (single-sided *P* = 0.02) (81).

Thromboprophylaxis with Certoparin; a LMWH, primarily active against factor Xa, was evaluated in the TOPIC-1 and TOPIC-2 studies that examined patients with advanced breast cancer (TOPIC-1) and stage III/IV non-small cell lung cancer (TOPIC-2), respectively (69). VTE occurrence was not different between the treatment groups in TOPIC-1 (4% treated with Certoparin vs. 4% receiving placebo, OR = 1.02, 95% CI, 0.3–3.48) and TOPIC-2 [which showed a non-significant trend toward efficacy in lung cancer group (4.5 vs. 8.3%, OR = 0.52, 95% CI, 0.23–1.12)].

More recently, Semuloparin, a novel ultra LMWH, was tested for its safety and efficacy in preventing VTE among patients with advanced solid cancers receiving chemotherapy (82). In a double-blind, multicenter trial, patients were randomly assigned to receive subcutaneous Semuloparin 20 mg once daily, or placebo. The primary efficacy outcome was the composite of any symptomatic DVT, any non-fatal PE, and VTE-related death. VTE occurred in 20 of 1,608 patients (1.2%) receiving Semuloparin, compared with 55 of 1,604 patients (3.4%) on placebo (HR: 0.36; 95% CI, 0.21–0.60). Major bleeding rates of patients receiving Semuloparin and placebo rates were similar, occurring in 1.2 and 1.1%, respectively (HR: 1.05; 95% CI, 0.55–1.99).

International guidelines do not recommend routine prophylaxis of cancer patients in the ambulatory setting who receive anti-neoplastic agents (5, 72–74). In this setting, thromboprophylaxis is only recommended for patients with Multiple Myeloma on Thalidomide or Lenalidomide-based combination chemotherapy. Table 3 summarizes the commonly used anticoagulant regimens for VTE prophylaxis.

**Table 3:** **Recommended anticoagulant regimens for venous thromboembolism prophylaxis and treatment in patients with cancer**.

Management	Drug	Regimen
**Prophylaxis**		
	Unfractionated heparin	5,000 IU SQ, every 8 h
	Dalteparin	5,000 IU SQ, daily
	Enoxaparin	40 mg SQ, daily
	Tinzaparin	2.5 mg SQ, daily
	Fondaparinux	75 IU/kg SQ, daily
**Initial treatment**		
	Unfractionated heparin	80 IU/kg IV bolus, then 18 IU/kg/h IV
	Dalteparin*	100 IU/kg SQ every 12 h or 200 IU/kg SQ every 24 h
	Enoxaparin*	1 mg/kg SQ every 12 h or 1.5 mg/kg SQ daily
	
	Tinzaparin	175 IU/kg SQ daily
	Fondaparinux*	<50 kg: 2.5–5 mg SQ, daily
		50–100 kg: 5–7.5 mg SQ, daily
		>100 kg: 7.5–10 mg SQ, daily
**Long-term treatment**		
	Dalteparin	200 IU/kg SQ daily × 1 month then 150 IU/kg SQ daily
	Warfarin	5–10 mg PO daily, adjust dose to INR 2.0–3.0

*Avoid in patients with creatinine clearance <35 ml/min or adjust dose based on anti-factor Xa level.

*Modified from Verso et al. (71)*.

### Special Considerations in Patients with Acute Leukemia and Hematopoietic Stem Cell Transplant Recipients

Patients with acute leukemia and hematopoietic stem cell transplant (HSCT) recipients present a special challenge in this regard as both groups are at great risk for both thrombotic and bleeding events. The incidence of VTE in patients with acute leukemia, for example, ranges from 1.7 to 12% (83). Thrombocytopenia experienced by many of these patients as a result of chemotherapy places them at great risk for bleeding complications. In one retrospective study, of 1,514 HSCT transplant recipients, 4.6% patients developed symptomatic VTE within 180 days of HSCT (84), while 3.6% of patients had had a fatal bleeding episode. Randomized clinical trials are required in order to devise the best regimen for VTE prophylaxis in this high-risk patient population.

## Treatment of Venous Thromboembolism

Venous thromboembolism treatment in cancer patients is no different from that of other medical patients. While diagnostic evaluation is underway, anticoagulation should be started in all patients in whom VTE is a serious consideration. Options for acute management include adjusted-dose UFH, fixed dose LMWHs or Fondaparinux. Options for chronic anticoagulation have been summarized in Table 3. If an oral Vitamin K antagonist (VKA) is chosen, initial overlap with a parenteral agent (e.g., UFH, etc.) is required for at least 5–7 days until the international normalized ratio (INR) is between 2 and 3 for at least 24 h. Studies have shown that in patients with advanced cancers, LMWHs significantly reduce the incidence of recurrent VTE (by as much as 50% in some studies) when compared to oral VKA, without any difference in bleeding complications (85–88). LMWHs also have the advantage of simplifying initial treatment, thus, making it feasible to manage VTE in the outpatient setting as well. According to international guidelines, LMWHs are preferable to full dose Warfarin for initial treatment of cancer-related VTE, particularly in the first 3–6 months (5, 72–74). In these patients, it is recommended that subsequent anticoagulant therapy with oral VKAs or LMWHs should be continued indefinitely or until the cancer has resolved. The safety and efficacy of LMWHs in cancer patients beyond a treatment duration of 6 months is unknown but currently under investigation.^3^

In an observational study, Kovacs et al. noted that upper extremity DVTs secondary to central catheters in cancer patients respond well to anticoagulation without removal of the catheter (89). Therefore, it is preferable in this setting to treat upper extremity DVT without removal of the CVC. Guidelines further recommend that anticoagulation should be continued for as long as the catheter is in place and for at least 3 months after its removal (90).

The significance of diagnosing and treating calf vein DVT has been questioned in the past (91, 92). In an observational study by Galanaud et al., no difference in the proportion of recurrent VTE between patients with distal DVT or isolated proximal DVT was detected (93). In a randomized study on patients with calf vein thrombosis, Lagerstedt et al. observed that progressive thromboembolism developed in 29% of the patients in the absence of 3 months of oral anticoagulation therapy (94). The NCCN guidelines recommend a minimum of 3 months of therapy until larger studies supporting a shorter treatment duration become available (95). Given the inherent risk of major and rarely fatal bleeding (96, 97), the NCCN guidelines recommend that thrombolytic therapy should be restricted to life- or limb-threatening thrombotic events (95).

Vena cava filter insertion is commonly performed for recurrent PE, extension of DVT while on anticoagulation, and in instances where anticoagulation is contraindicated. Their use, however, is not associated with any mortality benefit based on results from observational studies and large randomized controlled trials (9, 98–100). Additionally, there are serious safety concerns for complications, such as filter thrombus, embolization, fatal PE as well as fracture, and migration of retrievable filters (9, 101, 102). The NCCN guidelines, therefore, recommend that Vena Cava filters should only be used in the setting of acute VTE where a patient cannot receive anticoagulation.

A large randomized clinical trial evaluating thrombolytic therapy for sub-massive PE failed to show any mortality benefit (103). Additionally, there were concerns for bleeding complications with this form of therapy. The NCCN guidelines, therefore, recommend that use of systemic thrombolytics be reserved for massive PE. Recommendations for superficial venous thrombosis have been summarized in Table 4.

**Table 4:** **Therapeutic recommendations for venous thromboembolism in cancer**.

Deep vein thrombosis (DVT)	LMWH for acute and chronic therapy UFH, LMWH or Fondaparinux with transition over 5–7 days to Warfarin (INR 2–3) is an acceptable alternative if LMWH not feasible Duration at least 3 months or for as long as cancer active (whichever is longer) For massive DVT, consider catheter-directed pharmacomechanical thrombolysis If anticoagulation contraindicated, consider retrievable vs. permanent vena cava filter
Pulmonary embolism	LMWH for acute and chronic therapy UFH, LMWH or Fondaparinux with transition over 5–7 days to Warfarin (INR 2–3) is an acceptable alternative if LMWH not feasible Duration at least 6 months or for as long as cancer active (whichever is longer) For massive PE, consider thrombolytic therapy If anticoagulation contraindicated, consider retrievable vs. permanent vena cava filter
CVC-related DVT	Initial therapy with UFH, LMWH or Fondaparinux with transition over 5–7 days to Warfarin (INR 2–3) Remove catheter if symptoms fail to improve or catheter no longer needed Duration at least 3 months or for as long as catheter is present (whichever is longer) For massive CVC-related DVT consider thrombolytic therapy
Superficial venous thrombosis	If distal, consider symptomatic therapy with compresses, NSAID’s and continued observation If proximal (above knee), consider LMWH with or without transition to Warfarin (INR 2–3) particularly with clots within 2cm of deep venous system Duration of therapy 1–3 months
Calf vein thrombosis	Initial therapy with UFH, LMWH, or Fondaparinux with transition over 5–7 days to Warfarin (INR 2–3) or LMWH for acute and chronic therapy Duration of therapy 3 months If anticoagulation contraindicated, consider serial duplex surveillance If calf vein DVT progresses to involve proximal deep veins and anticoagulation is contraindicated, consider retrievable vs. permanent vena cava filter
Recurrent VTE	If currently on Warfarin switch to LMWH or treat for 5–7 days with UFH, LMWH, or Fondaparinux in transition to therapeutic INR (if INR sub therapeutic at time of event) If on LMWH- check dose, consider LMWH level vs. empiric dose increase, switch to Fondaparinux If recent initiation of UFH or LMWH, consider HIT Look for anatomic reason for recurrence Consider vena cava filter
Vena Cava filter	If a retrievable filter is placed, follow the patient closely and retrieve the filter when anticoagulation is no longer contraindicated If a permanent filter is placed and anticoagulation is no longer contraindicated, consider indefinite anticoagulation

*Adapted from Streiff (95)*.

Recurrent VTE despite adequate anticoagulation is not uncommon among cancer patients and an empiric approach has been proposed for such instances (104, 105). In all cases of symptomatic recurrent VTE, it is important to ensure drug compliance. Heparin-induced thrombocytopenia (HIT) must also be excluded. Patients on treatment with oral VKA should be switched to LMWH and those being managed with LMWH should have an increase in their dose by 25% (or increased to weight adjusted doses if receiving lower doses) (106, 107). In patients who do not respond, another dose escalation should be considered and an anti Xa level may be used to estimate the next dose escalation (108). Anatomic risk factors that may account for recurrent thrombosis (e.g., target vessel compression by tumor, May–Thurner syndrome, Thoracic outlet syndrome) should be excluded. Vena cava filter insertion may also be considered though this intervention has no impact on patient survival as described above.

### Bemiparin

Bemiparin is a LMWH with anti-factor Xa/anti-factor IIa activity that has been studied for VTE prophylaxis in cancer patients undergoing abdominal or pelvic surgery (109). In the CANBESURE study, 703 cancer patients undergoing surgery were randomized to receive 3,500 IU of Bemiparin subcutaneously daily for 8 days. They were then randomized to receive Bemiparin or placebo for an additional 20 days. Major VTE occurred in 0.8% of patients in the Bemiparin group compared with 4.6% in the placebo group (relative risk reduction 82.4%; 95% CI, 21.5–96.1%; *P* = 0.010). The study authors concluded that 4 weeks of Bemiparin use (when compared to 1 week of Bemiparin) significantly reduced the rate of major VTE without an associated increase in bleeding risk in cancer patients undergoing surgery.

### Semuloparin

Semuloparin is a subcutaneous ultra LMWH that acts as a factor Xa inhibitor with residual factor IIa activity (110). The TREK study evaluated the dose–response profile of Semuloparin in patients undergoing total knee replacement (110). A significant dose–response correlation was observed across five tested doses of Semuloparin and the incidence of VTE ranged from 5.3% (at a dose of 60 mg/day) to 44.1% (at a dose of 10 mg/day). A similar dose–response effect was observed for incidents of major bleeding (*P* = 0.0231).

It was concluded that a dose between 20 and 40 mg/day provides an adequate benefit-to-risk profile. Agnelli et al. evaluated Semuloparin for thromboprophylaxis in cancer patients receiving chemotherapy (82).

### Parenteral Factor Xa Inhibitors

Fondaparinux is a synthetic pentasaccharide that acts by binding to antithrombin and increasing its inhibitory effect against factor Xa by a factor of ~300. Turpie et al., in a meta-analysis of four multicenter, randomized, double-blind trials, evaluated the safety and efficacy of Fondaparinux in comparison with Enoxaparin (111). The incidence of VTE was reduced by half from 13.7% with LMWH to 6.8% with Fondaparinux. The US Food and Drug Administration has approved Fondaparinux as a substitute for Heparin or LMWH in the initial treatment of VTE. Additional studies are needed to evaluate the safety and efficacy of this agent in patients with malignancy.

### Oral Direct Factor Xa Inhibitors

Rivaroxaban (109, 112–115) and Apixaban (116–118) are two promising oral inhibitors of factor Xa that have been predominantly studied for prevention of VTE in patients undergoing orthopedic surgery.

In one study, extended duration prophylaxis with Rivaroxaban (10 mg once daily for 35 days) was compared with shorter duration prophylaxis with Enoxaparin (40 mg once daily for 10 days) in acutely ill medical patients, including patients with cancer (MAGELLAN trial) (119). The primary efficacy outcomes were symptomatic VTE, VTE-related death, or asymptomatic proximal DVT detected by routine compressive ultrasonography. Rivaroxaban was associated with a reduction in the risk of venous thrombosis compared to 10 days of treatment with Enoxaparin. Bleeding rates were significantly increased with this new factor Xa inhibitor.

In an interim analysis of a phase II trial, Levine et al. noted that Apixaban was well tolerated by patients with metastatic cancer. The incidence of major bleeding and thrombosis were very low among 125 patients (two patients receiving Apixaban at a dose of 20 mg and one patient in the placebo group developed major bleeding). Thrombosis was reported among three cases in the placebo group (120).

Edoxaban, another oral direct factor Xa inhibitor has been recommended as an alternative agent to treat VTE in cancer patients by the latest Chest guidelines (Grade 2C) (121). In an indirect comparison with apixaban, rivaroxaban, and dabigatran; edoxaban did not show statistically significant difference in risk of recurrent VTE or all – cause mortality (122). In addition, for the composite end – point of major or clinically relevant non-major bleeding, the relative risk for apixaban vs. edoxaban was 1.50 (95% CI, 1.17–1.92, *P* = 0.001), was 1.15 (95% CI, 0.95–1.39, *P* = 0.16) for rivaroxaban vs. edoxaban and 1.31 (95% CI, 1.02–1.68, *P* = 0.04) for edoxaban vs. dabigatran. However, this analysis did not look at the cancer patients. Therefore, edoxaban appears a safer alternative to Warfarin except patients with creatinine clearance >95 ml/min in whom it had shown to increase the risk of ischemic stroke. It does carry the benefit of once a day dosing as compared to its contemporary agents.

### Oral Direct Thrombin Inhibitors

Ximelagatran was the first oral direct thrombin inhibitor studied in clinical trials but its development was discontinued because of potentially severe hepatotoxicity. Dabigatran etexilate is the most developmentally advanced oral direct thrombin inhibitor. In randomized, double-blinded trials, it has been shown to be non-inferior to LMWH in reducing the risk of major VTE in patients undergoing orthopedic surgery.

## Conclusion

Thromboembolism in cancer patients is associated with significant consequences, including its association with morbidity, mortality, the need for long-term anticoagulation, and its consumption of healthcare resources. Recent studies have enabled us to better understand its clinical risk factors. New oral anticoagulants present an attractive treatment option because of their ease of (oral) administration and their lack of need for laboratory monitoring. Some of their limitations include:

(a) Published data on the safety and efficacy of NOACs in cancer-associated VTE is lacking. In clinical trials evaluating NOACs in VTE treatment, a very small percentage of the study population randomized to receive a NOAC had a diagnosis of cancer. Therefore, it remains unknown whether the safety and efficacy outcomes observed in these trials of largely non-cancer patients also apply to cancer patients.
(b) Recent trials comparing Fondaparinux or Rivaroxaban with LMWH have shown that specific factor Xa inhibition might be less efficacious than LMWH inhibition in cancer patients (123, 124).
(c) The oral administration route may not be practical in patients experiencing nausea, vomiting, and diarrhea as side effects of chemotherapy.
(d) No antidote is available to reverse the anticoagulant effects of these agents.
(e) The lack of assays to measure their anticoagulant effect makes it difficult to manage patients presenting with recurrent thrombosis or bleeding.
(f) Drug–drug interactions with anti-neoplastic agents may lead to clinically important changes in drug levels (105). The ISTH guidance statement, therefore, recommends against the use of NOACs for the treatment of cancer-associated thrombosis (108). It is imperative that the efficacy and safety of these agents be investigated in randomized controlled trials, specifically, in cancer patients with VTE.

## Author Contributions

The authors contributed to the idea, drafting, and main editing of the manuscript.

## Conflict of Interest Statement

All of the authors report no conflict of interest and have no biases based on industry, employment, consultancies, stock ownership, honoraria, paid expert testimony, or patent applications/registrations.

## Footnotes

^1^www.clinicaltrials.gov NCT 00876915

^2^www.clinicaltrials.gov No. NCT01602432

^3^www.clinicaltrials.gov, NCT00942968
